# In Silico Analyses Suggest That Exercise-Induced Irisin-Mediated Neuroprotection Supports Non-Pharmacological Preventive Strategies for Alzheimer’s Disease in Public Health

**DOI:** 10.3390/ijerph23040449

**Published:** 2026-04-01

**Authors:** Moara Manias, João Victor Rossetti Vieira, Aline Sampaio Cremonesi

**Affiliations:** 1 Department of Biomedical Sciences, São Francisco University, Bragança Paulista 12916-900, SP, Brazil; moara.manias@mail.usf.edu.br; 2 Laboratory of Computational Biology Applied to Proteins, Health Data Science Postgraduate Program, São Francisco University, Bragança Paulista 12916-900, SP, Brazil; joao.vieira@usf.edu.br

**Keywords:** bioinformatics, molecular docking, network proteins, neuroprotection, molecular dynamic

## Abstract

**Highlights:**

**Public health relevance—How does this work relate to a public health issue?**

- Alzheimer’s disease (AD) is the leading cause of dementia worldwide and imposes a significant economic and social burden on healthcare systems. In Brazil, expenditures on AD-related hospitalizations in the Unified Health System (SUS) exceeded R$ 12 million between 2020 and 2024, highlighting the urgent need for strategies to alleviate this financial and social burden.
- This study highlights how excess weight (overweight and obesity) in the Brazilian population is strongly correlated with increased hospitalizations for Alzheimer’s disease decades later. This directly connects the research to efforts in combating chronic non-communicable diseases and addressing the social and behavioral determinants of health.

**Public health significance—Why is this work of significance to public health?**

- The study presents physical exercise as a strategic public health tool, providing an epidemiological and molecular basis for the prevention of AD. As a low-cost and widely applicable intervention, exercise can be a viable alternative for promoting healthy aging on a large scale.
- By investigating irisin (an exercise-induced myokine) and its interaction with the *α*V/*β*5 receptor, the research provides evidence on how physical activity activates neuroprotective pathways and promotes neuronal plasticity. This offers scientific support for health policies to focus on prevention rather than solely on the treatment of established diseases.

**Public health implications—What are the key implications or messages for practitioners, policy makers and/or researchers in public health?**

- Promoting regular and supervised exercise should be a priority in public health policies to reduce the future incidence of dementia. Early investment in healthy habits can mitigate the impact of rapid population aging.
- The study also details the interaction interface between irisin and its receptor, paving the way for the development of novel molecules that mimic the effects of exercise. Furthermore, it underscores the importance of integrating epidemiological data from public systems (such as DATASUS) with bioinformatics tools to guide health interventions.

**Abstract:**

Alzheimer’s disease (AD) is the leading cause of dementia and imposes a high economic and social burden on healthcare systems. In Brazil, the consistent increase in costs associated with AD hospitalizations, coupled with the absence of curative therapies and population aging, reinforces the need for low-cost, broadly applicable preventive strategies. This study investigated the role of irisin, a myokine induced by physical activity, in the prevention of AD, integrating epidemiological and bioinformatic analyses. Public data on the nutritional status of the Brazilian population in the early 2000s and on AD hospitalizations approximately 20 years later were analyzed, assessing the temporal association using a lagged Spearman correlation. Additionally, genes associated with AD were analyzed through protein–protein interaction networks and functional enrichment. Structural models of irisin and the integrin *α*V/*β*5 receptor were employed in molecular docking and molecular dynamics analyses. Historical data indicated a high prevalence of excess weight in the early 2000s (46.7% ± 4.2% of the adult population) and a strong positive correlation with AD hospitalizations two decades later (ρ = 0.88; *p* = 0.033). Functional analyses revealed enrichment of pathways related to neurodegeneration, neurotrophins, and neuronal plasticity, involving proteins such as BDNF, AKT, ERK1/2, and CREB. Docking and molecular dynamics indicated a stable interaction of irisin with the *α*V/*β*5 receptor, suggesting activation of neuroprotective pathways. The findings reinforce physical exercise as a strategic public health tool for the prevention of AD, providing an epidemiological and molecular basis to reduce the future burden of the disease, thereby shifting the focus of public health policy from treatment to prevention.

## 1. Introduction

Alzheimer’s disease (AD) stands as one of the greatest contemporary challenges for global public health, being the leading cause of dementia and one of the most impactful chronic conditions associated with population aging. More than 55 million people were estimated to live with dementia in 2021, with a growing prevalence in people over 65 years of age and a greater impact among women, reflecting not only biological factors but also social and demographic determinants of the health-disease process [1]. The impact of AD transcends the clinical scope, imposing a high economic and social burden on healthcare systems. Globally, the cost of dementia was estimated at approximately $1.3 trillion USD in 2019 [2]. In the Brazilian context, between 2020 and 2024, expenditures on AD hospitalizations exceeded R$ 12 million, with an annual average of R$ 2.43 million, which represents a significant burden for the Unified Health System (SUS) [3]. DATASUS data also demonstrate a progressive increase in hospitalizations and mortality due to AD over the last decade, especially in women and individuals over 80 years of age [4]. These data indicate the need for strategies based on health promotion, prevention, and modulation of risk factors that are accessible and capable of positively impacting quality of life throughout the life course.

From a biological perspective, AD is characterized by the accumulation of *β*-amyloid (A*β*) plaques, formation of neurofibrillary tangles of hyperphosphorylated Tau protein, in addition to persistent processes of oxidative stress and neuroinflammation [1,5]. These mechanisms contribute to synaptic dysfunction, neuronal loss, and progressive cognitive decline. Although such molecular pathways are widely described, there is growing recognition that behavioral and environmental factors play a decisive role in modulating these pathophysiological trajectories [6]. Among the most associated modifiable factors for cognitive protection, physical exercise stands out. Evidence indicates that regular exercise practice is associated with reduced brain A*β* levels, decreased amyloid deposition, attenuation of neuroinflammation, stimulation of hippocampal neurogenesis, and improved cognitive performance, making it a promising strategy both in prevention and in maintaining cognitive function throughout aging [7].

Part of these benefits appears to be mediated by systemic physiological adaptations induced by exercise, including the release of peripheral factors capable of acting on the central nervous system such as irisin, a peptide derived from the cleavage of the FNDC5 (Fibronectin-type III domain-containing protein 5) protein, whose expression is stimulated by the activation of PGC-1*α* (Peroxisome proliferator-activated receptor-gamma coactivator) during physical exercise. Although initially described as a myokine, FNDC5 shows high expression in neurons, astrocytes, and microglia, also evidencing a relevant physiological role in nervous tissue [5]. Irisin is capable of crossing the blood-brain barrier and triggering responses associated with neural protection, including increased expression of BDNF (brain-derived neurotrophic factor), promotion of synaptogenesis, and greater neuronal survival [1]. Individuals with AD have reduced levels of irisin in the hippocampus and cerebrospinal fluid, while higher concentrations are associated with better cognitive function and lower amyloid pathology burden [1,5].

The irisin–BDNF axis has been proposed as one of the main mediators of the neuroprotective effects associated with exercise [6]. Suppression of FNDC5 expression reduces BDNF levels and compromises processes related to synaptic plasticity, neurogenesis, and memory [5]. BDNF, in turn, is reduced in patients with AD and its expression can be stimulated both directly by irisin and by associated intracellular pathways, such as cAMP/PKA/CREB and STAT3 [1,5]. Furthermore, irisin has been associated with the activation of neuroprotective signaling mechanisms, including Akt/ERK1/2, contributing to the reduction of oxidative stress and the inflammatory response [8]. There is also evidence that irisin may directly interfere with amyloid pathophysiology. Overexpression of FNDC5 reduces the secretion of A*β*40 and A*β*42, and its interaction with the amyloid precursor protein appears to modulate APP (Amyloid Precursor Protein) cleavage, contributing to lower production of neurotoxic peptides [9]. Experiments with recombinant irisin indicated its ability to attenuate oxidative stress induced by A*β* oligomers, reinforcing its potential role in neuroprotective signaling processes relevant to preventive strategies [8].

Recent advances have allowed the identification of the integrin *α*V/*β*5 receptor complex as the specific receptor for the central action of irisin, particularly in astrocytes. Suppression of the *β*5 subunit abolished the effect of irisin on reducing A*β* levels, indicating that this molecular interaction constitutes a critical functional axis [10]. Despite these advances, the structural and dynamic details of this interaction remain poorly understood, as well as the mechanisms that proceed from this interaction to neuroprotective effects, which limits the translational exploration of this mechanism as a basis for interventions inspired by the physiological effects of exercise.

The elucidation of the molecular mechanisms by which exercise promotes neuroprotection allows the development of public health strategies aimed at healthy aging and the prevention of chronic diseases. Thus, the present study investigated the role of irisin in the prevention of Alzheimer’s disease (AD) through the integration of epidemiological data from the Unified Health System (SUS) and bioinformatics tools, including protein-protein interaction networks, docking, and molecular dynamics. Such analyses substantiate the neuroprotective signaling of exercise at the molecular level, offering support for preventive strategies and interventions based on exercise physiology aimed at addressing cognitive decline and pathologies associated with aging.

## 2. Materials and Methods

### 2.1. Epidemiological Analysis with Time Lag

An ecological study with time-lagged series was conducted, based on secondary data from public access sources, aiming to investigate the association between nutritional status and the incidence of neurodegenerative diseases in the Brazilian population. The annual prevalence of overweight and obesity were extracted from the Food and Nutritional Surveillance System (SISVAN) for the period 2000 to 2007, used as the population exposure variable. Outcomes, comprising the annual volume of hospital admissions for neurodegenerative pathologies between 2020 and 2025, were obtained from Hospital Information System (SIH/SUS) via the DATASUS platform (TABNET) for the period 2020 to 2025. An approximate time lag of 20 years was adopted between the exposure (2000–2005) and outcome (2020–2025) periods. The correlation between excess weight prevalence and hospital admissions was assessed using Spearm’s coefficient in the RStudio environment (version 2026.01.0+392) [11], adopting a significance level of *p* < 0.05.

Ethical review and approval were waived for this study due to the exclusive use of secondary, aggregated, and anonymized publicly available data. In accordance with Resolution No. 510/2016 of the Brazilian National Health Council (CNS) (Art. 1, items II and III), research that uses information from the public domain that does not allow individual identification of participants is exempt from analysis by Ethics Committees [12].

### 2.2. Network Analysis

Genes associated with Alzheimer’s disease and physical exercise were selected from the GeneCards v5.2 database [13], following the association scores. Interactions with the FNDC5 protein were mapped via the STRING platform [14], using a medium confidence level or higher (score ≥ 0.6). The generated networks were processed in Cytoscape software (version 3.10.3) [15], where hub proteins were identified by the intersection of centrality metrics (degree, betweenness, and closeness) above the medians. Densely connected clusters were isolated using the MCODE algorithm (score ≥ 6) [16,17]. Functional enrichment analysis of the network proteins was conducted in DAVID [18], using pathways from the Kyoto Encyclopedia of Genes and Genomes (KEGG) database [19]. Significance was adjusted using the False Discovery Rate (FDR < 0.05) method, and magnitude was expressed by fold enrichment. Causal interactions, including directionality and regulatory effects, were extracted from the SIGNOR 4.0 database [20]. These data were integrated to construct a directed signaling network, aiming to identify regulatory modules associated with neuroprotection and neuronal plasticity.

### 2.3. Structural and Molecular Dynamics (MD) Analyses

The primary sequences of irisin (FNDC5) and integrins *α*V and *β*5 were obtained from the KEGG database and processed on the DeepTMHMM server [21] to identify signal peptides and transmembrane domains. Signal peptides were removed prior to structural modeling in ColabFold [22], an optimized implementation of the AlphaFold2 algorithm [23]. Models with a pLDDT score ≥ 90 were selected; structures with lower values underwent refinement in GalaxyRefine [24]. Stereochemical validation was conducted via GDT-HA, RMSD, MolProbity score, Clash score metrics, and Ramachandran plot analysis. The *α*V/*β*5 heterodimer was constructed by structural alignment in ChimeraX [25], using the template PDB ID: 3VI3. Protonation states were adjusted to pH 7.4 on the PDB2PQR server [26] with the PARSE force field. Protein-protein docking was performed in ClusPro 2.0 [27] using the rigid-body method. Conformations were ranked by binding free energy, prioritizing complexes with the highest affinity. The interaction interface was analyzed to identify critical residues and intermolecular contacts, using a distance cutoff of up to 3.0 Å.

The most energetically favorable complex was subjected to Molecular Dynamics (MD) simulations in GROMACS software (version 2025.1) [28], via the Visual Dynamics (version 4.2.1) platform (Fiocruz) [29]. The system was solvated in an octahedral box with the Simple Point Charge (SPC) water model and a minimum wall distance of 1.0 nm, using the CHARMM27 force field. After minimization and equilibration, the 5 ns trajectory was monitored for structural stability and conformational fluctuations through RMSD, RMSF, and Radius of Gyration (Rg) metrics.

## 3. Results

### 3.1. Epidemiological and Network-Based Analysis of the Association Between Overweight and Alzheimer’s Disease

Analysis of DATASUS data revealed that, between 2000 and 2007, the average prevalence of excess weight (overweight and obesity) in the Brazilian adult population was 46.17% (±4.2%). The time-lagged analysis evidenced a strong and statistically significant positive correlation between nutritional status in the early 2000s and the volume of hospitalizations for dementia and Alzheimer’s disease two decades later (ρ = 0.94, *p* = 0.016 and ρ = 0.88; *p* = 0.033, respectively). Although it does not indicate causality, the correlation supports the hypothesis that long-term excess weight may be a determining risk factor for neurodegeneration within the context of the Brazilian public health system.

**Figure 1:**
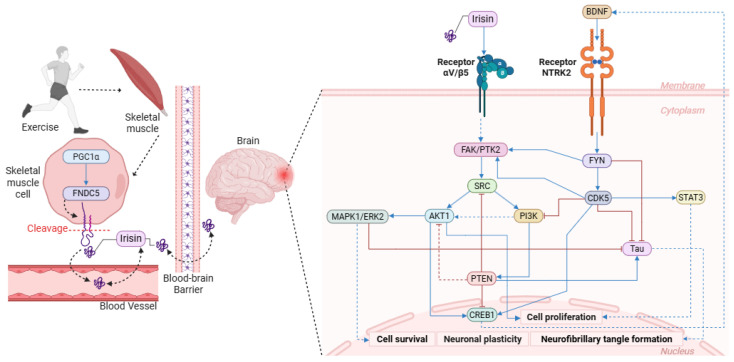
Schematic representation of the production, migration, and mechanisms of action of irisin induced by physical exercise. The network was constructed based on functional enrichment (DAVID), protein–protein interactions (SIGNOR 4.0), and the literature. Irisin, produced in skeletal muscle, is released into the circulation, crosses the blood–brain barrier, and reaches the brain, where it can interact with *α*V/*β*5 integrin receptors expressed on astrocytes. This interaction is associated with the activation of FAK/PTK2 and SRC, with subsequent activation of the PI3K/AKT pathway, related to cell survival and proliferation and neuronal plasticity; the PKA/CREB pathway, which increases BDNF expression and hippocampal cell proliferation; and the MAPK/ERK pathway, which causes blockade of Tau protein, responsible for the formation of neurofibrillary tangles characteristic of Alzheimer’s disease. Solid blue arrows indicate direct upregulated interactions; dashed blue arrows represent indirect interactions; red arrows with flat ends indicate downregulated. Created in Biorender. Moara Manias. (2026) https://BioRender.com.

From a set of 11,695 genes associated with Alzheimer’s disease identified in GeneCards, it was observed that the FNDC5 protein establishes direct interactions with 22 protein targets involved in various biological processes (Table 1). Expanding the network to a second level of interaction totaled 753 proteins, which were subjected to functional enrichment analysis. This process revealed 210 distinct biological pathways, of which 207 showed statistical significance (FDR ≤ 0.05). Among the overrepresented categories, the Neurodegeneration–Multiple Diseases pathway (Fold Enrichment = 5.77) and the Neurotrophin pathway (Fold Enrichment = 7.03) stood out, the latter already described in the literature as a possible target of irisin [1]. It is known that irisin is a peptide hormone, derived from the cleavage of FNDC5 whose expression is induced by the action of PGC-1*α* (Figure 1) [30,31]. Irisin crosses the blood-brain barrier and modulates gene expression associated with neuroprotection in the hippocampus, such as that of the BDNF protein, promoting neurogenesis and neuronal cell survival [1].

The network modeling based on the *α*V/*β*5 receptor found in astrocytes was also performed at two levels of expansion, resulting in a network of 755 proteins and revealing an association with the PI3K-Akt pathway (Fold Enrichment = 19.67), recognized as a central regulator of cellular metabolism, growth, proliferation, and survival. Although the magnitude of the Fold Enrichment may be influenced by the restricted number of proteins associated with these pathways, the data suggest that the *α*V/*β*5 receptor complex acts in the modulation of these biological processes (Figure 1), with potential intensification of its activity in the presence of irisin.

According to our analyses, irisin signaling occurs via interaction with receptors of the integrin family, specifically the *α*V/*β*5 complex, through which it exerts its biological effects and activates the AKT and ERK protein pathways, which in brain tissue have been associated with mediating the neuroprotective effect [32]. Experimental evidence demonstrates that chemical inhibition of these pathways results in suppression of neuroprotective benefits, reinforcing the dependence of these mechanisms for neuronal preservation [33]. Another mechanism associated with AD that was also identified as a target of irisin action was the formation of neurofibrillary tangles (NFTs) associated with Tau protein hyperphosphorylation. Proteins associated with irisin stimulation promote Tau inhibition, reducing the formation of NFTs associated with synaptic loss and memory impairment [1]. It was also observed that irisin is not currently annotated in pathways deposited in the KEGG and Signor 4.0 databases, evidencing the limitation of systematized information on its molecular mechanisms of action and reinforcing the need for studies aimed at elucidating its signaling pathways in the context of neuroprotection.

### 3.2. Structural Analysis of the Irisin-*α*V/*β*5 Receptor Interaction and Conformational Stability of the Complex

The structure of monomeric irisin was obtained from the Protein Data Bank (PDB ID: 4LSD), and the N-terminal portion of each integrin was modeled via ColabFold and individually refined using GalaxyRefine. The models presented adequate quality parameters for the analyses (Table 2). For the assembly of the *α*V/*β*5 complex, the structure deposited under PDB code 3VI3 was used as a basis.

**Figure 2:**
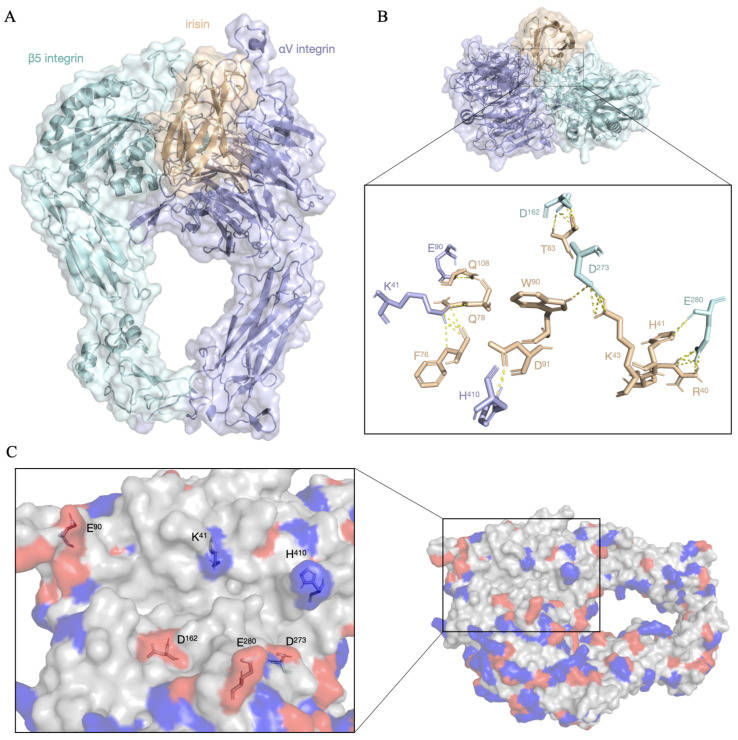
Representation of the structure of the *α*V/*β*5-irisin complex and the amino acid residues involved in the interaction. (**A**) Representation of the formation of the *α*V/*β*5-irisin complex. (**B**) Top view of the complex highlighting the amino acid residues identified as possibly involved in the interaction with a maximum distance of 3.0 Å. (**C**) Protein surface colored according to the acidic–basic character of the amino acid residues. Blue regions correspond to basic residues, red regions to acidic residues, and white regions to neutral residues. An acidic groove formed by residues D162, D273, and E280 of *β*5 integrin, and a basic groove formed by residues K41 and H410 of *α*V integrin can be identified.

Molecular docking simulations suggest a stable interaction interface between irisin in its monomeric form and the *α*V/*β*5 complex (Figure 2A), consistent with the structural model previously described by Mu and colleagues [33]. The interaction exhibited an average binding free energy value of −808.07 kcal/mol, with a standard deviation of 45.48 kcal/mol, suggesting high affinity between the molecules. Complex stabilization occurs predominantly through electrostatic interactions, involving acidic residues of the *α*V and *β*5 integrins and basic residues of the *α*V integrin (Figure 2C). In the *α*V subunit, residues E90 interacting with Q108 of irisin, K41 with residues F76 and Q78, and H410 with D91 stand out. In the *β*5 subunit, interactions were observed between residue D162 with T83 of irisin, E280 with R40 and H41, and D273 with K43 and W90, establishing a set of specific bonds with distances below 3.0 Å (Figure 2B) that contribute to complex stability.

**Figure 3:**
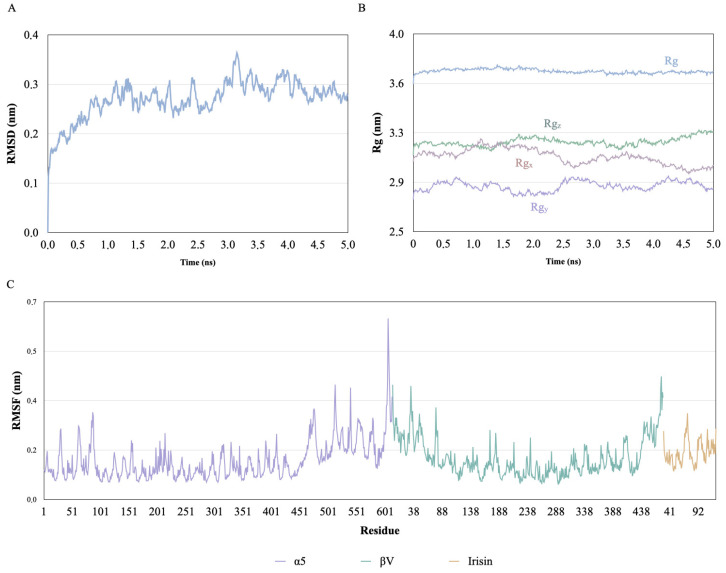
Molecular dynamics analysis of the *α*V/*β*5-irisin integrin complex. (**A**) RMSD indicates instability at the initial moment of interaction followed by stabilization of the complex after 3.0 ns of interaction. (**B**) Total radius of gyration of the complex (Rg), and on each axis (x, y, and z), indicating variations over time characteristic of the interaction. (**C**) RMSF of the complex residues with specific fluctuation points and greater portion of stability.

RMSD (Root Mean Square Deviation) analysis during the molecular dynamics indicated initial instability in the first picoseconds of the simulation, followed by stabilization indicating the consolidation of the interaction between irisin and the *α*V/*β*5 receptor (Figure 3A). The radius of gyration remained globally stable; however, a distinct variation was observed in the Cartesian axes starting at 3 ns. There was an increase in Y-axis values accompanied by a reduction in the X-axis, suggesting a conformational elongation of the complex during the interaction (Figure 3B).

For the analysis of local flexibility, residues located within 10 positions of the N and C-termini were excluded. To ensure a statistically sound characterization of local flexibility and mitigate distortions caused by outliers, the Median Absolute Deviation (MAD) was used. MAD is recommended for data from non-Gaussian distributions in biomolecular systems and is recognized as a robust metric for analyzing variability in molecular dynamics [34,35]. The correction factor rho was calculated as σ = 1.4826 × MAD, and the fluctuation values were converted into standardized scores using the robust z-score = (RMSF_i_ − median RMSF)/σ. Residues with a robust z-score ≥ 2 or above the 95th percentile were classified as highly mobile. Additionally, a rolling average with a five-residue window was applied to smooth out point-specific oscillations and highlight structural trends related to conformational dynamics. The *α*V residue H410, although flexible, did not fall within the zones of highest structural mobility, while irisin residue R71 was located in a region of high flexibility (Figure 3C), consistent with the intrinsically mobile loops of the protein.

The average solvent-accessible surface area (SASA) per residue was evaluated, and the stability of this exposure was estimated by the coefficient of variation throughout the trajectory. SASA analysis revealed that the residues involved in the interaction interface remained predominantly exposed to the solvent throughout the simulation, including those located in the acidic and basic grooves of the receptor, which facilitates interaction with irisin. The mean coefficient of variation was 0.36, corroborating the RMSF data, indicating stability of the residues, despite the high solvent accessibility.

## 4. Discussion

### 4.1. The Neuroprotective Mechanisms of Irisin in Brain Health and Alzheimer’s Disease

The observed association between excess weight in the early 2000s and the increase in Alzheimer’s disease (AD) cases in subsequent decades is consistent with the hypothesis of a plausible temporal relationship between metabolic factors and neurodegenerative outcomes. However, this interpretation should be made with caution. The lack of public data prior to 2000 regarding the nutritional status of the Brazilian population limits the number of time points and reduces the statistical power of the ecological analysis. The inclusion of more recent periods is not methodologically acceptable because the population analyzed comprises adult individuals and requires a time interval compatible with the latency of neurodegenerative diseases. Shorter intervals could compromise the biological plausibility of the outcomes, considering that part of the individuals would not yet have reached the age groups in which AD incidence becomes more significant. Nevertheless, relevant confounding factors remain: (I) increased life expectancy in recent decades, considering that age is the main risk factor for AD [36]; (II) socioeconomic factors, such as education and access to health services, which can simultaneously influence the prevalence of excess weight and the risk of cognitive decline [36]; and (III) advances in diagnosis, which may have contributed to the increased identification of cases [37]. Underreporting should also be considered because AD diagnosis is not mandatory notifiable in Brazil [38] and is often not recorded as the primary cause of hospitalization in hospital systems. Despite these limitations, the observed correlation is consistent with evidence associating higher body mass index with increased dementia risk, especially when obesity occurs in midlife [36]. This scenario is reinforced by the high prevalence of excess weight already identified in Brazil in the early 2000s, which directly impacts public finances [39]. Furthermore, the consistent increase in childhood obesity in Brazil, with a prevalence of 12.2%, suggests an additional risk factor for the future development of non-communicable chronic diseases, including neurological conditions associated with aging [36,40]. In combination with population aging and the high burden of modifiable risk factors, these data support the relevance of AD as an emerging public health problem.

The influence of modifiable factors is also evident in different socioeconomic contexts. In high-income countries, better conditions of education, nutrition, and access to healthcare are associated with a reduction in dementia incidence, although recent trends such as increased obesity and sedentary behavior may reverse this scenario. In contrast, low-income countries concentrate the majority of dementia cases, which can be explained both by increased life expectancy and by the higher prevalence of modifiable risk factors, such as hypertension, obesity, and low education [36]. Habits associated with weight reduction and quality of life have already been reported as ways to prevent mental disorders in older adults. Pereira and colleagues [41] analyzed 85 Brazilians aged between 60 and 80 years and identified an inverse association between a healthy lifestyle, lower body mass index, and lower prevalence of common mental disorders. These studies reinforce that even in the older population, lifestyle plays a relevant role in maintaining body weight and mental health quality.

Among the main components of a healthy lifestyle, regular physical exercise stands out, contributing not only to body weight control but also through direct effects on the central nervous system. Irisin, derived from FNDC5 cleavage, is one of the main molecular mediators of these effects [6]. Evidence indicates that activation of the PGC-1*α*/FNDC5/irisin axis is associated with increased expression of brain-derived neurotrophic factor (BDNF), modulation of inflammatory processes, and promotion of neuronal plasticity [6,41]. These effects are consistent with the findings of the network analysis, which demonstrated significant functional enrichment (FDR ≤ 0.05) in pathways such as AMPK, PI3K/AKT, PKA/CREB, and ERK1/2, all previously associated with neuroprotection. These pathways have complementary functions. While AKT and ERK1/2 proteins promote neuronal protection by reducing pro-inflammatory cytokines such as tumor necrosis factor (TNF)-*α* [5], the PKA/CREB pathway regulates the expression of BDNF related to cell proliferation in the hippocampus and neurogenesis via STAT3 signaling [1]. Additionally, it has been observed that BDNF expression is induced by irisin-mediated cAMP signaling but is reduced in AD patients [8]. These signaling cascades are also related to decreased neuronal apoptosis, reduced aggregation of *β*-amyloid (A*β*) peptide, and modulation of amyloid precursor protein (APP) cleavage, including the reduction of the C99 C-terminal fragment directly associated with A*β* formation [6,32,33,42]. Tau protein inhibition pathways were also identified, which is relevant considering its central role in the formation of neurofibrillary tangles, considered a core event in the pathophysiology and progression of AD [1].

The identification and characterization of these pathways, together with clinical evidence that obese individuals with a family history of Alzheimer’s disease have reduced serum irisin levels compared to non-obese individuals [43], reinforce the plausibility of its neuroprotective role and position physical exercise as a non-pharmacological alternative for the prevention of dementias such as AD. Thus, public policies aimed at promoting regular physical activity can generate long-term benefits, contributing to reducing the burden of neurological and chronic diseases in the public health system.

### 4.2. Structural Mechanisms Associated with the Irisin–*α*V/*β*5 Interaction

The structural characterization of the interaction between irisin and the *α*V/*β*5 integrin receptor should be interpreted considering the limitations inherent to integrin studies, especially the absence of complete experimental structures of the receptor–ligand complex. Nevertheless, the mean free energy of −808.07 kcal/mol is compatible with a favorable binding affinity, consistent with the formation of a stable complex. This value supports the hypothesis that the interaction between irisin and *α*V/*β*5 represents an initial event for the activation of previously described intracellular pathways [44]. Molecular docking analysis is consistent with the interaction being predominantly stabilized by a network of electrostatic contacts involving residues located within the acidic and basic grooves. This same profile was observed in a previous experimental model [44] and reinforces the relevance of these domains for ligand molecular recognition and subsequent signal transduction. Although there were discrepancies between the residues possibly involved in the complex interaction in our model when compared to the experimental model, this may be related to differences in the structural refinement protocols employed, as well as to the dynamic nature of integrins. Integrin–ligand interaction is known to induce global and local conformational changes in the receptor [45,46], which may justify the structural discrepancies observed between studies. Cryo-EM structures demonstrate that the *α*IIb*β*3 complex undergoes a swing-out movement that can reach up to 70°, reflecting increased ligand-induced structural flexibility [45]. Complementarily, X-ray crystallography and Nuclear Magnetic Resonance (NMR) studies have identified the transition between open (high-affinity) and closed (low-affinity) conformational states, a process known as the “switchblade” model [46]. It is known that *α*V/*β*5 integrins interact with their ligands through recognition of the RGD (Arg-Gly-Asp) motif [46,47]; however, irisin does not possess the RGD motif and binds to the *α*V/*β*5 receptor at an alternative site located on the opposite face of the RGD pocket, without competing with traditional ligands such as fibronectin 10 [10,44], which was also observed in our simulation. This characteristic suggests that irisin-mediated signaling may activate intracellular pathways distinct from other ligands of the same receptor, corroborating previous studies [10].

Molecular dynamics simulations indicated global stability of the complex, as evidenced by RMSD. Initial structural movements of approximately 0.2 nm were observed within the first 4.0 ns, possibly associated with conformational adjustments resulting from irisin binding. These structural adjustments are consistent with the literature, which describes displacements on the order of 0.1 nm in lateral helices of the *α* subunit after induction of the open state [46]. Such behavior is characteristic of molecular recognition systems, in which complex formation reduces the conformational entropy of the isolated proteins [48]. Changes in the radius of gyration (Rg) axes after 3 ns suggest a possible induced-fit mechanism, compatible with the optimization of irisin docking into the acidic and basic grooves of the receptor. RMSF analysis using robust statistical metrics (MAD and z-score) allowed for the discrimination of thermal fluctuations from potentially functionally relevant movements. Residue R71 exhibited high mobility in irisin, consistent with conformational flexibility compatible with exploring the receptor surface before complex stabilization. In contrast, residue H410 of the *α*V subunit displayed moderate flexibility, possibly relating to a more stable structural role in ligand anchoring. The variations observed in solvent-accessible surface area (SASA) indicate that despite exposure to the aqueous environment, the complex maintains structural stability. This pattern suggests that the interaction is predominantly sustained by electrostatic and geometric complementarity rather than by the formation of a classical hydrophobic core, a characteristic often observed in transient interactions associated with cell signaling processes [45].

The epidemiological, network, and structural data are consistent with a plausible model in which excess weight acts as a modifiable risk factor for AD, whereas physical activity exerts a protective effect, at least in part, through irisin-mediated mechanisms. Accordingly, strategies aimed at reducing excess weight and promoting physical activity across the lifespan should be considered a public health priority. Considering the multifactorial nature of AD and the presence of modifiable risk factors, early population-based interventions may have a significant impact on reducing the future burden of the disease, especially in contexts with high obesity prevalence and limitations in epidemiological surveillance systems. On the other hand, although the activation of multiple intracellular signaling pathways associated with irisin is widely documented in the literature, the network analysis performed in this study did not identify a direct intracellular partner of the *α*V/*β*5 integrin complex responsible for transmitting these signals. This absence should not be interpreted as evidence of the biological non-existence of the interaction, but possibly as a reflection of limitations inherent to the databases and algorithms used in network construction, which rely on previously recorded and curated interactions. However, some studies indicate that Focal Adhesion Kinase (FAK/PTK2), identified in the networks of the present study, may act as an initial mediator of signal transduction [44,47], although this connection was not explicitly recovered by the tools employed. This gap highlights the need for further investigations aimed at identifying molecules and protein complexes that connect the receptor to the activation of downstream pathways, representing a critical axis for the comprehensive understanding of irisin’s mechanisms of action and for advancing therapeutic strategies based on this system.

## 5. Conclusions

The high rate of excess weight observed in the Brazilian population since the early 2000s may be associated with the increase in Alzheimer’s incidence approximately 20 years later. Although the analysis is limited by factors such as underreporting, a small number of time points, and potential confounders, the findings are consistent with the literature and reinforce the relevance of metabolic factors in modulating dementia risk. Network analyses suggest that irisin is associated with the activation of intracellular pathways involved in neuroprotection, synaptic plasticity, and inflammatory regulation. Together, these findings support the hypothesis that regular physical activity may contribute to the long-term prevention of dementia in the population.

Structural analyses demonstrated that the interaction of irisin with the *α*V/*β*5 receptor complex is stabilized by specific contacts with acidic and basic grooves on the receptor, in agreement with previously described evidence. Molecular dynamics simulations indicate that this interface is maintained by a balance between structurally stable electrostatic interactions and regions of conformational changes, favoring efficient ligand recognition and stabilization of the receptor–ligand complex even in the absence of the RGD domain present in integrin interaction partners. The detailed characterization of this interaction interface provides a mechanistic basis for the rational development of new molecules capable of selectively modulating the *α*V/*β*5 receptor located on astrocytes, directing specific molecular responses with functional and physiological consequences. This approach represents a widely employed strategy in the development of new drugs, particularly in the context of neurodegenerative diseases, in which the modulation of neuroprotection and synaptic plasticity pathways constitutes a relevant therapeutic target.

The results converge on an integrated model in which lifestyle factors, particularly those related to weight control and physical activity, may influence AD risk through specific molecular mechanisms. Although they do not establish causality, the findings reinforce the relevance of preventive interventions at the population level, especially in contexts with a high prevalence of modifiable risk factors.

## Figures and Tables

**Table 1:** Proteins identified with direct interaction with irisin (FNDC5) via the STRING platform, organized according to their main functions. Proteins are represented by gene names, as stated in databases such as KEGG and UniProt.

Name	Function
Cellular Protection and Survival	
AKT1	Regulate cell proliferation, survival, metabolism, and angiogenesis in both normal and malignant cells.
BDNF	Promotes neuronal survival and synaptic plasticity in the adult brain.
CTSB	Amyloid precursor protein secretase involved in APP proteolytic processing.
CRP	It recognizes pathogens and damaged host cells and initiates their elimination.
FN1	Fibronectin is involved in cell adhesion and migration processes.
TGFBR2	Receptor mediating TGF-*β* signaling and proliferations control.
TNF	Proinflammatory cytokine regulating proliferation, differentiation, immune and apoptotic pathways.
Metabolism and Hormones	
ADIPOQ	Produced in adipose tissue and is involved with metabolic and hormonal processes.
GHRL	It is a powerful appetite stimulant and plays an important role in energy homeostasis.
INS	It plays a vital role in the regulation of carbohydrate and lipid metabolism.
LEP	It regulates energy homeostasis by acting on the brain.
PPARG	Nuclear receptor regulating adipogenesis and lipid metabolism.
SHBG	Transports androgens and estrogens in the blood.
Inflammatory Processes	
IL6	Proinflammatory cytokine involved in immune regulation.
IL1B	Key mediator of inflammatory response, cell proliferation, differentiation and apoptosis.
Mitochondrial Activity	
HSPA4	Molecular chaperone assisting protein folding and stability.
PPARGC1A	Transcriptional coactivator regulating mitochondrial biogenesis.
TFAM	Mitochondrial transcription factor controlling mDNA replication and repair.
UCP2	Mediates proton leak and thermogenesis, regulating metabolic efficiency.
Cellular Growth	
IGF1	Growth factor promoting cell proliferation and survival.
PPARA	Nuclear receptor regulating fatty acid metabolism.
Interaction with DNA	
SIRT1	It regulates epigenetic gene silencing and suppresses recombination of rDNA.

**Table 2:** Parameters of the refined models of *α*V/*β*5 integrins by GalaxyRefine. GDT-HA and MolProbity are dimensionless metrics. ClashScore is expressed as the number of steric clashes per 1000 atoms.

Integrin	GDT-HA	RMSD (Å)	MolProbity	Clash Score	Poor Rotamers (%)	Rama Favored (%)
*α*V	0.9918	268	1.543	10.5	1.0	98.9
*β*5	0.9884	283	1.624	13.0	0.7	98.5

## Data Availability

Epidemiological data were obtained from the publicly available DATASUS platform (https://datasus.saude.gov.br (accessed on 16 February 2026)). All structural data used in this study are included within the article. Data generated from molecular docking and molecular dynamics simulations are available from the authors upon reasonable request.
